# Impact of Maturity of Malay Cherry (*Lepisanthes alata*) Leaves on the Inhibitory Activity of Starch Hydrolases

**DOI:** 10.3390/molecules22060873

**Published:** 2017-05-24

**Authors:** Yan Zhang, Yuyang Gui, Dejian Huang

**Affiliations:** 1 Food Science and Technology Programme, c/o Department of Chemistry, National University of Singapore, 3 Science Drive 3, Singapore 117543, Singapore; zhang_yan@u.nus.edu (Y.Z.); yuyang2511@gmail.com (Y.G.); 2 National University of Singapore (Suzhou) Research Institute, 377 Lin Quan Street, Suzhou Industrial Park, Suzhou 215123, Jiangsu, China

**Keywords:** Malay cherry leaves, wheat noodles, rice noodles, in vitro study, digestibility

## Abstract

Aqueous extracts of young (7-day-old) Malay cherry (*Lepisanthes alata*) leaves were incorporated into wheat and rice flours to evaluate their inhibitory activities against α-amylase and α-glucosidase. HPLC-ESI/MS^2^ results showed that the active components in young leaves were proanthocyanidins with lower mean degrees of polymerisation (≤10). The IC_50_ of the aqueous extracts of young leaves were 2.50 ± 0.03 and 12.91 ± 0.29 µg/mL, against α-amylase and α-glucosidase, which make them less active compared to the mature leaves. In contrast, total proanthocyanidins in aqueous extracts decreased as the leaves matured, indicating that the compounds in the mature leaves have much higher activity. However, there was no significant difference in the digestibility of wheat noodles incorporated with the aqueous extracts from either young or mature leaves. Interestingly, with regard to rice noodles, their digestibility was mostly reduced by incorporating aqueous extracts of young leaves compared to using mature leaves.

## 1. Introduction

Blood glucose is mainly contributed by dietary carbohydrates such as starch, which are enzymatically hydrolysed by salivary and followed by pancreatic α-amylase into oligosaccharides, and then finally by intestinal α-glucosidase into glucose before they can be taken up by the small intestine [1]. Maintenance of our blood glucose level within the normal range is crucial for our health, especially for Type-II diabetic patients with postprandial hyperglycemia [2]. Consumption of high glycemic index (GI) foods (GI ≥ 70) leads to a sharp increase in blood glucose levels, and thus consumption of low GI foods (GI ≤ 55) is recommended for Type-II diabetic patients by reducing the postprandial blood glucose level [3]. The demand for starchy staple foods with low GI increases with the increasing population of Type-II diabetic patients. Therefore, starchy staple foods incorporated with starch hydrolase inhibitors are designed to reach low GI by delaying glucose release and absorption rates and thereby reducing postprandial hyperglycemia. Presently, some α-glucosidase inhibitors are taken as oral anti-diabetic medications (e.g., acarbose, voglibose, and miglitol) with strong adverse effects (e.g., undesirable gastrointestinal symptoms) and thereby not suitable for food application. These adverse effects are due to the excessive loading of fermentable sugars that is being transported to the large intestine where intestinal microbes produce gas by fermentation [4]. To eliminate these adverse effects, the use of α-amylase inhibitors would be desirable so as to reduce the amount of fermentable sugars [5].

Noodles have been consumed as daily staple foods since ancient time in Asia and have become increasingly popular in other parts of the world. Approximately up to 50% of the total wheat is consumed by Asians as noodles [6]. Moreover, rice noodles are broadly consumed in Southeast Asia and gaining popularity [7]. However, most of noodles are considered as high GI foods [3] and not friendly to consumers with the need of reduced absorption of carbohydrates for controlling blood glucose or body weight. Incorporation of α-amylase inhibitors, extracted from edible plants, into noodles would render the noodles with low GI.

Our previous study discovered that the aqueous extracts of Malay cherry mature leaves showed 8.5 times more potency than acarbose in inhibiting α-amylase and that the active components are proanthocyanidins. The proanthocyanidins exhibited a mean degree of polymerisation (mDP) at 27 and were composed of (epi)catechins and (epi)gallocatechins linked through B-type interflavanyl bonds [8]. Based on our previous results, Malay cherry is an extremely promising edible plant containing α-amylase inhibitors that can act as antidiabetic ingredients in functional or medical food systems, thereby meeting the increased demand of Type-II diabetic consumers. We incorporated aqueous extracts of Malay cherry mature leaves into wheat, rice, tapioca, and potato flours with the purpose to develop noodles with slow digestibility. The digestibility of tapioca noodles (excluding protein) incorporated with proanthocyanidins equivalents (0.3%) of mature leaves’ aqueous extracts is maximumly reduced by 69.2%, whilst the digestibility of wheat noodle (including protein) is minimumly reduced by 30.6%. These results are probably due to the interference of proanthocyanidin-protein binding affinities. Therefore, we suspected that proanthocyanidins from young leaves might have weaker proanthocyanidin-protein binding affinities than those from mature leaves, because the mDP of proanthocyanidins is a major determinant of its affinity to proteins, and more highly polymerised proanthocyanidins have stronger affinities for proteins [9]. It is known that the mDP of polyphenols (including proanthocyanidins) in plants is positively correlated with their age and maturity [10,11].

As a plant develops and matures, polyphenols are continuously polymerised so that high molecular weight polymers are built up at the expense of their monomers and oligomers and eventually become dominant in the plant [12]. In light of these findings, we suspect that proanthocyanidins from Malay cherry young leaves would have lower degree of polymerisation and may behave differently comparing to mature leaves, as active ingredients for noodles with lower GI. Therefore, the objectives of our study were to: (1) investigate the changes of the mDP of proanthocyanidins, inhibitory activity against starch hydrolases, and total proanthocyanidins during the maturity process of Malay cherry leaves; and (2) evaluate the digestibility of noodles incorporated with aqueous extracts of Malay cherry young and mature leaves.

## 2. Results and Discussion

### 2.1. Analysis of Total Proanthocyanidins

Total proanthocyanidins in aqueous extracts were quantified by the DMAC assay, which involves the condensation of the nucleophilic site of the phloroglucinol structure (A-ring) of proanthocyanidins with DMAC to form colored complexes detectable at 640 nm [13].

The total proanthocyanidins in aqueous extracts of young leaves (115.88 ± 5.95 mg procyanidin A2 equivalent/g aqueous extracts) showed a significant (*p* < 0.05 by *t*-test) higher amount as compared to that of mature leaves (37.59 ± 2.55 mg procyanidin A2 equivalent/g aqueous extracts). These results suggested that the total proanthocyanidins in the aqueous extracts were significantly reduced as the leaves matured. The decreasing trends in total proanthocyanidins were in agreement with several studies conducted on leaves of various plants: mangrove [14], blackberry, raspberry, strawberry [15], and mangrove [14], respectively. Proanthocyanidins are a subclass of phenolics. The greater production of proanthocyanidins in young leaves could be attributed to the fact that they are required to provide structural support for photosynthesis and defense strategy against invasion by pathogenic microbes and being consumed by insects and herbivores [16]. Also, the higher rates of metabolite biosynthesis in young tissues may account for the higher level of proanthocyanidins in young leaves [17]. As the leaves matured, the demand for proanthocyanidins decreases [14]. Moreover, proanthocyanidins condense from monomers to oligomers as the leaves matured [12]. DMAC prefers the C8 of the A-ring and reacts only with the terminal units of proanthocyanidins [13]. That’s one reason for the above decreasing trend.

Despite the fact that total proanthocyanidins of aqueous extracts of mature leaves are much lower than that of young leaves, the starch hydrolase inhibitory activities of the former are stronger than that of the latter. This suggests that there is no direct correlation between total proanthocyanidins and starch hydrolase inhibitory activities. Instead, inhibitory activities are mainly influenced by the specific structure of proanthocyanidins [18].

### 2.2. Characterisation of Proanthocyanidins Using HPLC-ESI/MS^2^ and MALDI-TOF/MS

**Figure 1:**
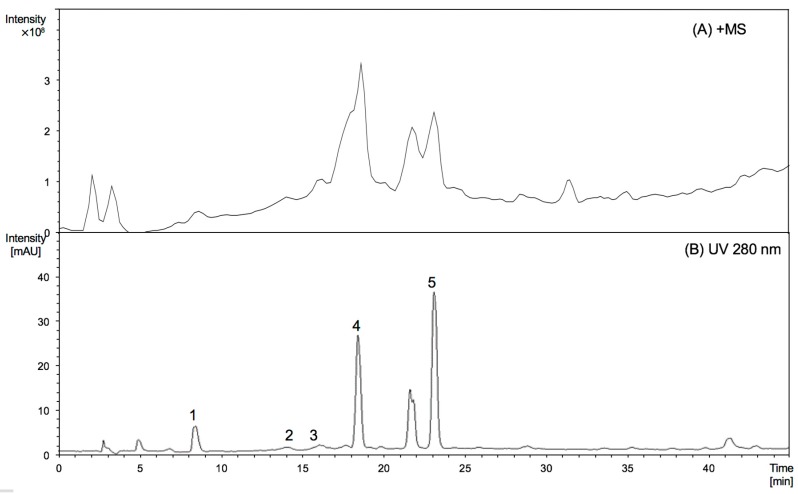
MS spectrograms (**A**, positive mode) and HPLC chromatograms (**B**, 280 nm) of thiolysed products of proanthocyanidins from Malay cherry young leaves. Peaks (1) epigallocatechin; (2) (epi)gallocatechin thioether; (3) epicatechin; (4) (epi)gallocatechin thioether; and (5) (epi)catechin thioether are shown.

To characterise the nature of the extension units of proanthocyanidins, thiolysed proanthocyanidins from Malay cherry young leaves were analysed by HPLC-ESI/MS^2^ (Figure 1 and Table 1).

The peaks numbered from 1 to 5 detected in young leaves were identified as epigallocatechin, (epi)gallocatechin thioether, epicatechin, (epi)gallocatechin thioether, and (epi)catechin thioether [8]. From the HPLC profile, it is obvious that the young leaves were identical to mature leaves. Figure 1 further shows that (epi)gallocatechin thioether and (epi)catechin thioether were the major products of thiolysed proanthocyanidins, which suggests that there were significant amounts of (epi)gallocatechin and (epi)catechin extension units in proanthocyanidins from young leaves, while (epi)catechins are the major component of the extension units from mature leaves. By comparing the peak areas of the individual peaks obtained from HPLC chromatogram, the mDP of proanthocyanidins from young leaves was estimated to be 10, quite different from the mDP of proanthocyanidins described previously for mature leaves (mDP at 27). This increasing trend of mDP values is in line with literature reports on other leaves [10,11].

**Figure 2:**
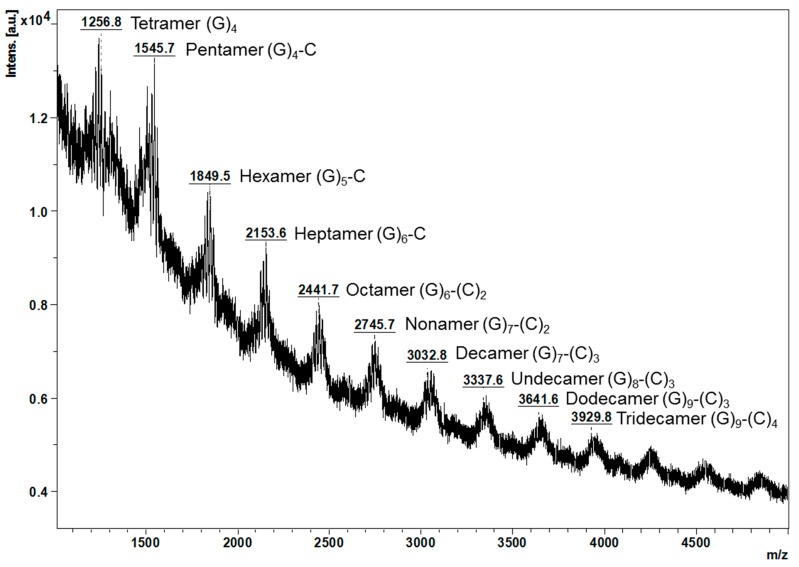
Observed masses of proanthocyanidins from Malay cherry young leaves by MALDI-TOF/MS. C and G are the abbreviations for (epi)catechin and (epi)gallocatechin, respectively. The stereochemistry of the chiral carbons on the C ring of flavanols units is not defined. All the interflavanyl bonds are B-type. All mass-to-charge ratio (*m*/*z*) refers to [M + K]^+^.

MALDI-TOF/MS spectra of the proanthocyanidins from young leaves further verified the thiolysis results (Figure 2). It can be observed that ions below *m*/*z* 2745.7 were more intense, which suggests that proanthocyanidins from young leaves were slightly polymerised, in agreement with the mDP at 10. A series of masses at *m*/*z* [M + K]^+^ which could correspond to oligomeric proanthocyanidins ranging from tetramer (*m*/*z* 1256.8) to tridecamer (*m*/*z* 3929.8) were noted. The difference in mass between every two adjacent peaks was observed to be *m*/*z* 288 or 304, which matches the mass of (epi)catechin or (epi)gallocatechin, respectively.

Considering for example the ions below *m*/*z* 2745.7, the ion at *m*/*z* 1256.8 might be a tetramer composed only of (epi)gallocatechins, the ion at *m*/*z* 1545.7 could correspond to a pentamer of four (epi)gallocatechins and one (epi)catechin, the ion at *m*/*z* 1849.5 could be a hexamer of five (epi)gallocatechins and one (epi)catechin, the ion at *m*/*z* 2153.6 might be a heptamer of six (epi)gallocatechins and one (epi)catechin, and the ion at *m*/*z* 2441.7 could correspond to a octamer of six (epi)gallocatechins and two (epi)catechins. In contrast, the proanthocyanidins from the mature leaves only display one major peak at *m*/*z* 4409.7 and were tentatively assigned to a pentadecamer of three (epi)gallocatechins and twelve (epi)catechins [8].

### 2.3. Inhibitory Activities against α-Amylase and α-Glucosidase

**Figure 3:**
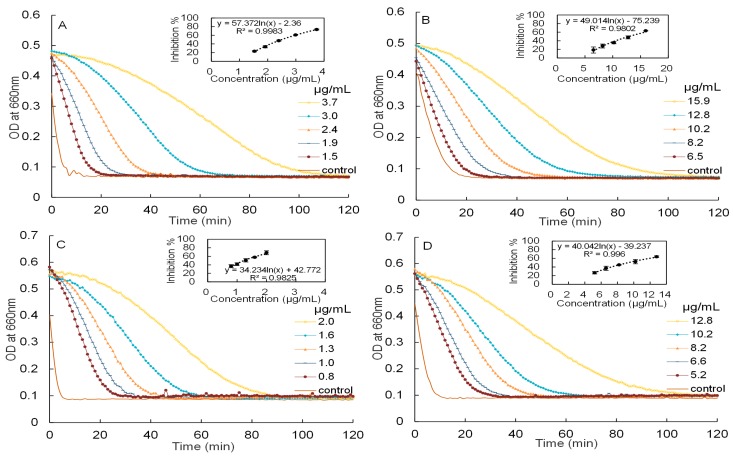
The kinetic curves of starch hydrolysis in the presence of different concentrations and corresponding dose response curves of starch hydrolase inhibitory activity. (**A**) aqueous extracts of Malay cherry young leaves and (**C**) proanthocyanidins against α-amylase are shown; (**B**) aqueous extracts of Malay cherry young leaves and (**D**) proanthocyanidins against α-glucosidase are shown.

Inhibitory activities of mature leaf extracts against α-amylase and α-glucosidase have been reported in our previous paper [8]. In this study, the inhibitory activities of young leaf extracts against α-amylase and α-glucosidase will be investigated, as showed in Figure 3.

The IC_50_ values of the aqueous extracts of young leaves against α-amylase and α-glucosidase were measured to be 2.50 ± 0.03 and 12.91 ± 0.29 μg/mL, respectively. These are significantly (*p* < 0.05 by ANOVA) weaker than the IC_50_ of the purified proanthocyanidins of young leaves against α-amylase and α-glucosidase (1.24 ± 0.10 and 9.30 ± 0.55 μg/mL, respectively). As a reference, the IC_50_ of acarbose, measured under the same conditions, were 6.56 (for α-amylase) and 2.12 μg/mL (α-glucosidase), respectively. Therefore, the aqueous extracts and proanthocyanidins of young leaves were approximately 2.6 and 5.3 times more potent than acarbose in inhibiting α-amylase, while the aqueous extracts and proanthocyanidins of mature leaves were close to 8.5 and 11.5 times more potent than acarbose. For convenient comparison, the IC_50_ of young leaf extracts was further expressed as acarbose equivalent (AE). The aqueous extracts and proanthocyanidins of young leaves had inhibitory activities of 4.1 mmol AE/g and 8.2 mmol AE/g against α-amylase, whilst had inhibitory activities of 0.3 mmol AE/g and 0.4 mmol AE/g against α-glucosidase, respectively. Moreover, the aqueous extracts and proanthocyanidins of Malay cherry leaves exhibited a stronger inhibitory activities against α-amylase as compared to α-glucosidase. The trend obtained is in line with studies conducted on proanthocyanidins derived from chiku [18] and *Vaccinum floribundum* [19]. Being a more potent inhibitor against α-amylase as compared to α-glucosidase, proanthocyanidins of Malay cherry leaves can potentially be used to selectively target α-amylase and not α-glucosidase in the treatment of diabetes, and this would reduce the adverse effects associated with excessive loading of fermentable sugars caused by high levels of α-glucosidase inhibitory activity.

Besides, a methanol fraction obtained from the Sephadex LH-20 column was tested for its inhibitory activities against α-amylase and α-glucosidase and it showed little activity at 0.17 mg/mL and 0.26 mg/mL, respectively. This was expected as elution using methanol would serve to wash away the impurities present in the aqueous extracts of young leaves from the Sephadex LH-20 column. The thiolysed proanthocyanidins (1.0 mg/mL) were tested for their inhibitory activities; the absence of detectable inhibitory activities for both α-amylase and α-glucosidase suggests that polymerised proanthocyanidins were likely responsible for potent inhibitory activities and were not their monomers or other compounds in the young leaves.

The inhibitory activities of proanthocyanidins against starch hydrolases were observed that there was an increasing trend as the Malay cherry leaf matured. Therefore, we hypothesize that mature leaf proanthocyanidins, due to their higher mDP, would exert stronger inhibitory activities against starch hydrolases as compared to young leaf proanthocyanidins. Other studies corroborated our hypothesis. Li et al. found that proanthocyanidins from peel of *Choerospondias axillaris* fruit exhibited stronger inhibitory activity against α-amylase and α-glucosidase as the mDP increased [20]. Lee, Cho, Tanaka, and Yokozawa found that polymeric proanthocyanidins from persimmon exhibited a stronger inhibitory activity against α-amylase as compared to their oligomeric counterparts [21]. In conclusion, there exists a positive correlation between the mDP and inhibitory activities against starch hydrolases of proanthocyanidins.

### 2.4. In Vitro Digestibility Analysis of Noodles

**Figure 4:**
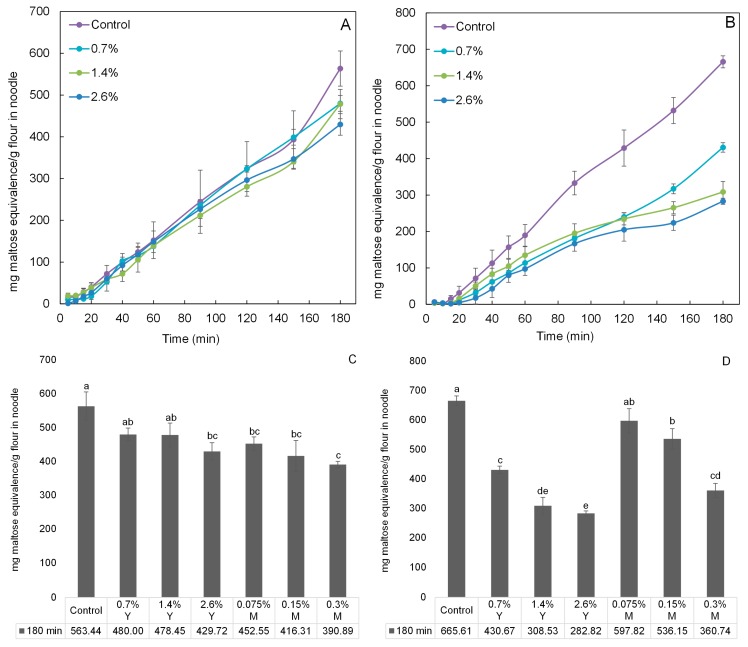
Digestibility expressed as mg maltose equivalence/g flour in noodle during in vitro digestibility analysis. The digestibility analysis of (**A**) wheat noodles and (**B**) rice noodles incorporated with aqueous extracts of Malay cherry young leaves are shown (during 180 min). The digestibility analysis of (**C**) wheat noodles and (**D**) rice noodles incorporated with aqueous extracts of Malay cherry young and mature leaves are shown (at 180 min). Significant differences (*p* < 0.05) for means in each plot were noted using different letters. Y and M are the abbreviation for aqueous extracts of Malay cherry young and mature leaves, respectively.

The digestion profiles of wheat and rice noodles incorporated with proanthocyanidins equivalents of 0%, 0.7%, 1.4%, and 2.6% for young leaves’ aqueous extracts were investigated using an in vitro digestibility analysis (Figure 4). The digestibility generally increased with time. Highest digestibility was observed for the control for both noodle types since α-amylase activity was at its highest in the absence of its inhibitors. The incorporation amounts increasing from 0.7% to 2.6% generally led to an increase in α-amylase inhibitory activity, as reflected by the decreasing digestibility of both noodle types, respectively. With respect to the control at 180 min, the effectiveness of proanthocyanidins from young leaves (from 0.7% to 2.6%) in slowing down starch digestion seems to be greater when incorporated into rice noodles as compared to wheat noodles (Figure 4C,D). This result may be due to the higher protein content of wheat flour (11%) than that of rice flour (5%), thereby generating stronger proanthocyanidin-protein binding affinity. In this study, only young leaves were investigated and the results of mature leaves cited from our previous study [22]. As shown in Figure 4C, wheat noodles incorporated with aqueous extracts from either young or mature leaves with the same inhibitory activity showed insignificant differences (*p* > 0.05) in the digestibility at 180 min. For instance, there was no significant difference between the digestibility of wheat noodle incorporated with 0.7% proanthocyanidins equivalents of young leaves and that with 0.075% proanthocyanidins equivalents of mature leaves. Based on results of mDP of proanthocyanidins discussed earlier, mDP of young leaf proanthocyanidins was estimated to be 10 and mDP of mature leaf proanthocyanidins was at 27. Young leaf proanthocyanidins should had weaker proanthocyanidin-protein binding affinity than that of mature leaf proanthocyanidins because proanthocyanidins with lower mDP have weaker affinities for proteins [9]. Therefore, the digestibility of wheat noodles incorporated with aqueous extracts of young leaves should be significantly lower than that of wheat noodle incorporated with the same inhibitory activity of aqueous extracts of mature leaves. The absence of this significant difference might be attributed to the fact that the wheat flour has a relatively high protein content of 11% (as compared to 5% in the rice flour), and most of the young and mature leaf proanthocyanidins were bound by these proteins through the formation of hydrogen bonds and hydrophobic interactions [9]. In conclusion, in order to significantly (*p* < 0.05) lower the digestibility of wheat noodles (the control), the minimum incorporation amount of young leaves was 2.6%, whilst that of mature leaves was only 0.075%. The digestibility of wheat noodle incorporated with 2.6% proanthocyanidins equivalents of young leaves was reduced by 23.73% at 180 min, compared to the control.

In Figure 4D, the digestibility of rice noodles incorporated with young leaves were significantly (*p* < 0.05) lower than that of rice noodle incorporated with the same inhibitory activity of mature leaves at 180 min. For example, the digestibility of rice noodle incorporated with 0.7% proanthocyanidins equivalents of young leaves was significantly lower than that of rice noodle with 0.075% mature leaves. One reason might be that the higher mDP of mature leaf proanthocyanidins enhanced their effectiveness in proanthocyanidin-protein binding [9,23]. Moreover, the fact is that less proteins were present in rice flour (5%), and hence a greater amount of free and uncomplexed young leaf proanthocyanidins were found in rice noodles (as compared to those in wheat noodles). Another reason might be attributed to the difference in total proanthocyanidins of young and mature leaves’ aqueous extracts. To maintain the inhibitory activity of aqueous extracts of young and mature leaves at the same level, proanthocyanidins equivalents of 0%, 0.7%, 1.4%, and 2.6% for young leaves’ aqueous extracts and 0%, 0.075%, 0.15%, and 0.3% for mature leaves’ aqueous extracts were mixed thoroughly with wheat and rice flour, respectively. Therefore, it is obviously found that proanthocyanidin equivalents of young leaves’ aqueous extracts was approximately 9 times higher than that of mature leaves’ aqueous extracts. That is the reason why the digestibility of rice noodles incorporated with young leaves were significantly lower than that with the same inhibitory activity of mature leaves. As can be seen also from Figure 4D, the digestibility of rice noodles (the control) can be significantly lower with minimum incorporation amount (0.7%) of young leaves at 180 min, while the minimum incorporation amount of mature leaves was up to 0.15%. The digestibility of rice noodle incorporated with 2.6% young leaves was maximumly reduced by 57.51% at 180 min, compared to the control. The stable thermal stability of proanthocyanidins was observed in the previous study. The phenolic compounds including proanthocyanidins were heated at 150 °C for 20 min and they showed minor losses (<10%) [24]. Compared with our heat processing (100 °C for 5 min), loss of proanthocyanidins would occur, but not too much.

## 3. Materials and Methods

### 3.1. Materials

Corn starch, α-amylase (type VI-B, from porcine pancreas), α-glucosidase (in the form of rat intestine acetone powder), pepsin (from porcine gastric mucosa), acarbose, 3,5-dinitrosalicylic acid (DNSA), Rochelle salt (potassium sodium tartrate), potassium carbonate, methyl thioglycolate, 2,5-dihydroxybenzoic acid, 4-dimethylaminocinnamaldehyde (DMAC), and procyanidin A2 (HPLC; purity >99%) were purchased from Sigma-Aldrich (St. Louis, MO, USA). Calcium chloride was obtained from Thermo Fisher Scientific Inc. (Waltham, MA, USA). Hydrochloric acid (37%), analytical grade solvents used for leaf extraction and Sephadex LH-20 were obtained from Merck (Darmstadt, Germany). HPLC grade solvents for preparation of mobile phases were purchased from VWR International GmbH (Darmstadt, Germany). Acetic acid (glacial) was purchased from RCI Labscan (Bangkok, Thailand). Sodium carbonate was purchased from GCE Chemicals (Malmö, Sweden). Phosphate buffered saline was purchased from Vivantis Technologies (Subang Jaya, Malaysia). Plain wheat flour (carbohydrates 71.1%, proteins 11.0%, fats 1.2%, and dietary fiber 2.9%) was purchased from Prima Ltd. (Singapore). White rice flour (carbohydrates 80.0%, proteins 5.0%, and fats 1.3%) was obtained from Bob's Red Mill Natural Foods, Inc. (Milwaukee, OR, USA). Fine salt was obtained from NTUC Fairprice Cooperative Ltd. (Singapore). Malay cherry leaf sprout (between 0.5 and 1.5 cm) at the top of leaf stalks was tagged on 2 October 2015 in Singapore. The first harvesting of young leaves (7-day-old) was made 7 days after the tagging. Leaf lengths of young leaves (7-day-old) varied between 5 and 7 cm.

### 3.2. Extraction, Purification and Characterisation of Proanthocyanidins from Young Leaves of Malay Cherry

Malay cherry young leaf powder (200 g) was freeze-dried and extracted with deionised water (1:3, *w*/*v*) by shaking on a vortex shaker (Rotamax 120, Heidolph Instruments GmbH & Co.KG, Schwabach, Germany) at 200 rpm for 12 h. The slurry was centrifuged (Eppendorf 5810R, Iberica, Madrid, Spain) at 12,074 g and 4 °C for 10 min to get the supernatant. The supernatant was condensed at 40 °C using a rotary evaporator (R-200, Büchi Labortechnik AG, Flawil, Switzerland) and then freeze-dried to give the aqueous extracts (25 g).

The resulting aqueous extracts from young leaves were used for purification of proanthocyanidins by Sephadex LH-20 column (100 mL, GE Healthcare BioSciences AB, Uppsala, Sweden) with the isolation yield of 1.5%. Briefly, the column was washed with 500 mL of 50% methanol, and the eluate (labelled as methanol fraction) was collected for subsequent analysis. The adsorbed proanthocyanidins were subsequently eluted with acetone (100 mL, 70%). The acetone was removed via rotary evaporation at 40 °C and the resulting aqueous solution was freeze-dried to give proanthocyanidins.

The HPLC-ESI/MS^2^ and MALDI-TOF/MS analyses and profiling of proanthocyanidins as well as thiolysis analysis for extension units of proanthocyanidins were also carried out according to our previous paper [8]. In brief, the proanthocyanidins (250 μL, 2 mg/mL in methanol) were mixed with acidified methanol (250 μL, 3.3% HCL in methanol, *v*/*v*) and methyl thioglycolate (500 μL, 5% in methanol, *v*/*v*). The mixture was heated at 45 °C for 0.5 h to complete the thiolysis reaction and injected into the HPLC-ESI/MS^2^ system with a reversed-phase C18 Sunfire column (250 mm × 4.6 mm i.d., 5 μm; Waters, Wexford, Ireland). The mobile phase A was 2% acetic acid in deionised water and mobile phase B was methanol. The thiolysed proanthocyanidins was monitored at 280 nm using elute gradient from 15% B to 80% B in 45 min at 1.0 mL/min. ESI/MS^2^ was acquired using a Bruker Amazon ion trap mass spectrometer (Billerica, MA, USA) equipped with a Dionex ultimate 3000RS LC system (Bannockburn, IL, USA) with a full scan mass spectra from *m*/*z* 100–2000 in both positive and negative ion modes. MALDI-TOF/MS was performed on a Brucker microTOF-QII mass spectrometer (Bruker Daltonics, Bremen, Germany). The proanthocyanidins (1 µL, 10 mg/mL in water) was mixed with 2,5-dihydroxybenzoic acid matrix (4 µL, 20 mg/mL in water). The dried mixture was introduced into the spectrophotometer with a positive polarity and linear flight path.

### 3.3. Quantification of Total Proanthocyanidins

The total proanthocyanidins of the sample was quantified according to the method reported by Prior et al. with slight modifications [25]. To each well of a 96-well plate, 70 μL of aqueous extracts obtained from young or mature leaves was added, and followed by addition of 210 μL of DMAC solution, which was made up of 0.1% (*w*/*v*) DMAC reagent in acidified ethanol (ethanol (91%): deionised water:HCl (36%), 75:12.5:12.5, *v*/*v*/*v*). The dilution solution was made by mixing ethanol (91%) with deionised water (4:1, *v*/*v*). The reaction mixtures were incubated at 25 °C and the absorbance was measured at 640 nm using a microplate reader (Synergy HT; Biotek Instruments Inc., Winooski, VT, USA) with one reading every minute for 30 min. The maximum absorbance readings obtained during the 30 min readings were used to determine total proanthocyanidins of the sample using the procyanidin A2 calibration curve of *y* = 24.303*x* + 0.0151 with *R*^2^ = 0.997, where y represents the absorbance at 640 nm and x represents the concentration of procyanidin A2 in mg/mL. Results were expressed as milligram procyanidin A2 equivalence per gram aqueous extracts.

### 3.4. Inhibitory Activities against α-Amylase and α-Glucosidase

The inhibitory activities of aqueous extracts and proanthocyanidins from Malay cherry young leaves was assessed by the turbidity assay according to Liu and co-workers [26]. Briefly, 20 µL of the serial-diluted sample or acarbose or buffer (as control) was pre-incubated with α-amylase (20 µL, 2 U/mL) or α-glucosidase (20 µL, 1 × 10^−2^ U/mL) at 37 °C for 15 min in the microplate reader. The mixture was mixed with gelatinised corn starch solution (60 μL, 20 mg/mL) and monitored at 660 nm for 2 h. Sodium phosphate buffer (0.1 M, pH 6.9) was freshly prepared with calcium chloride (40 ppm).

### 3.5. Preparation of Wheat and Rice Noodles with Reduced Digestibility

To maintain the inhibitory activity of aqueous extracts of young and mature leaves at the same level, proanthocyanidin equivalents of 0%, 0.7%, 1.4% and 2.6% for young leaves’ aqueous extracts and 0%, 0.075%, 0.15%, and 0.3% for mature leaves’ aqueous extracts were mixed thoroughly with wheat and rice flour, respectively. Formulated wheat flour was separately followed by addition of salt (0.2 g) and deionised water (6.3, 6.5, 6.6, or 6.8 mL) to form wheat flour doughs. The prepared wheat flour doughs were allowed to rest at room temperature for 10 min before making into noodles (2.0 mm in thickness and 6.0 mm in width) using a pasta machine (Marcato Atlas 180; Marcato S.p.A., Campodarsego, Italy). Formulated rice flour was then added with 15 mL of deionised water to get rice flour slurries, respectively, followed by equilibration for 1 h. Rice flour slurry (10 mL) was spread evenly on a stainless steel tray and then steamed for 5 min to form cooked rice noodle sheets (2.0 mm in thickness), followed by cooling for 30 min before making into noodles (6.0 mm in width) using the pasta machine.

### 3.6. In Vitro Digestibility Analysis of Noodles

Noodle (500 mg) was added to 5 mL buffer in a centrifuge tube. The wheat noodle was cooked by boiling in the buffer for 5 min followed by cooling to room temperature (this step was skipped for rice noodle since it was already cooked by steaming). The pH of the content was adjusted to 1.5 with 1 M HCl, after which it was incubated with 3.5 mg of pepsin (3200 U/mg) for 1 h in water bath (37 °C, 150 rpm). After pepsin incubation, the pH of the content was readjusted to 6.9 with 1 M K_2_CO_3_ before it was transferred to dialysis tubing (Snakeskin^TM^). Amylase (69 mg, 16 U/mg) was then added and each tubing was placed in a bottle containing 100 mL buffer. The bottle and its content was then allowed to incubate in water bath (37 °C, 150 rpm) for 3 h. Aliquots (300 μL) of the dialysate were removed at 0, 5, 10, 15, 20, 30, 40, 50, 60, 90, 120, 150, and 180 min with replacement for quantification of reducing sugars using the DNSA assay. 100 μL of dialysate mixed with 100 μL of DNSA reagent and then added to the wells of a 96-well plate, which was heated in boiling water for 5 min. After cooling to room temperature, 100 µL of the mixture was pipetted from each well and transferred into the corresponding wells of a new 96-well microplate and measured at 540 nm using the microplate reader. The absorbance was further calculated to maltose equivalence using the maltose calibration curve. Digestibility (the amount of reducing sugars released) was then expressed as milligram maltose equivalence per gram flour in noodle using Equation (1):

(1) mg maltose equivalence/g flour in noodle=maltose concentration (mg/mL) × 100 ÷ flour (g) × dough or slurry (g) ÷ 0.5

where, maltose concentration is calculated using the maltose calibration curve; dough or slurry refers to wheat dough or rice slurry; and 100 and 0.5 refer to the constant buffer volume (mL) and cooked noodle weight (g) for in vitro digestibility analysis, respectively.

### 3.7. Statistical Analysis

All analyses were performed in triplicate and results were expressed as mean ± standard deviation. Statistical analyses were performed using a one-way analysis of variance (ANOVA) with Tukey post-hoc multiple comparison test and an independent sample *t*-test in IBM SPSS Statistics V.22.0 (IBM Corporation, Armonk, NY, USA). Statistical significance was set at *p* < 0.05.

## 4. Conclusions

In conclusion, the in vitro results presented in this study revealed no significant differences (*p* > 0.05) in the digestibility of wheat noodles incorporated with the same inhibitory activity of aqueous extracts from young and mature leaves. However, digestibility of rice noodles incorporated with young leaves were significantly (*p* < 0.05) lower than that of rice noodles incorporated with the same inhibitory activity of mature leaves at 180 min. In addition, the mDP of proanthocyanidins, which increased from approximately 10 in young leaves to 27 in mature leaves, was found to be positively correlated with the maturity of the Malay cherry leaves. Inhibitory activities of aqueous extracts and proanthocyanidins against starch hydrolases were found to be age-dependent and increased as the leaves matured. Total proanthocyanidins in aqueous extracts significantly decreased as the leaves matured.

## Figures and Tables

**Table 1:** ESI/MS^2^ of thiolysed products of proanthocyanidins from Malay cherry young leaves.

RT (min)	Peak No.	Compound	MW	[M − H]^−^ (*m*/*z*)	MS^2^ Main Fragments (*m*/*z*)	[M + H]^+^ (*m*/*z*)	MS^2^ Main Fragments (*m*/*z*)
9.3	1 *	Epigallocatechin	306	305	179	307	139
14.0	2	(Epi)gallocatechin thioether	410	409	303	411	305, 287
14.7	3 *	Epicatechin	290	289	245, 205	291	271
18.7	4	(Epi)gallocatechin thioether	410	409	303	411	305, 287
23.1	5	(Epi)catechin thioether	394	393	287	395	289, 271

* Peaks 1 and 3 were identified using authentic standards.
